# Weakly supervised spatial relation extraction from radiology reports

**DOI:** 10.1093/jamiaopen/ooad027

**Published:** 2023-04-22

**Authors:** Surabhi Datta, Kirk Roberts

**Affiliations:** School of Biomedical Informatics, The University of Texas Health Science Center at Houston, Houston, Texas, USA; School of Biomedical Informatics, The University of Texas Health Science Center at Houston, Houston, Texas, USA

**Keywords:** information extraction, relation extraction, weak supervision, data programming, natural language processing, radiology report

## Abstract

**Objective:**

Weak supervision holds significant promise to improve clinical natural language processing by leveraging domain resources and expertise instead of large manually annotated datasets alone. Here, our objective is to evaluate a weak supervision approach to extract spatial information from radiology reports.

**Materials and Methods:**

Our weak supervision approach is based on data programming that uses rules (or labeling functions) relying on domain-specific dictionaries and radiology language characteristics to generate weak labels. The labels correspond to different spatial relations that are critical to understanding radiology reports. These weak labels are then used to fine-tune a pretrained Bidirectional Encoder Representations from Transformers (BERT) model.

**Results:**

Our weakly supervised BERT model provided satisfactory results in extracting spatial relations without manual annotations for training (spatial trigger F1: 72.89, relation F1: 52.47). When this model is further fine-tuned on manual annotations (relation F1: 68.76), performance surpasses the fully supervised state-of-the-art.

**Discussion:**

To our knowledge, this is the first work to automatically create detailed weak labels corresponding to radiological information of clinical significance. Our data programming approach is (1) adaptable as the labeling functions can be updated with relatively little manual effort to incorporate more variations in radiology language reporting formats and (2) generalizable as these functions can be applied across multiple radiology subdomains in most cases.

**Conclusions:**

We demonstrate a weakly supervision model performs sufficiently well in identifying a variety of relations from radiology text without manual annotations, while exceeding state-of-the-art results when annotated data are available.

## INTRODUCTION

Fine-grained clinical information extracted from radiology reports can be used in various downstream applications including large-scale medical image annotation,^1^^,^^2^ cohort retrieval,^3^ and automated tracking of findings.^4^ However, creating a large enough labeled dataset is pivotal for efficiently utilizing advanced deep neural models. In this work, we adapt a weak supervision approach—data programming—to automatically create a large labeled dataset of radiology reports for spatial relation extraction. We further validate the advantage of applying a pretrained language model on the weakly labeled reports, achieving satisfactory performance without hand-labeled training data.

Most information extraction studies in the clinical domain utilize exclusively supervised learning approaches. Such approaches rely on human-annotated reports that are not only tedious, time-consuming, and expensive, but also require extensive domain knowledge. Thus, it is difficult to achieve the scale of manual annotation for complex and fine-grained information. Moreover, manual annotations are often not generalizable across institutions because of limited coverage of language variations and/or reporting style. Meanwhile, deep learning-based supervised methods often demand large amounts of annotated training data to achieve substantial performance improvement over alternatives like rule-based methods. Recent research^5–8^ has proposed weak supervision to address the above issues by programmatically creating very large training corpora with imperfect labels which have the potential to outperform fully supervised approaches. Such approaches have been applied in clinical natural language processing (NLP) tasks such as medical entity classification,^9^ concept normalization,^10^ relation classification,^11^^,^^12^ and sentence-level classification^13^ for different use cases including patient-centered outcome assessment^13^ and medical device surveillance.^12^

One recently explored weak supervision method is data programming,^5^ which uses multiple supervision sources including patterns, domain rules, and domain-specific ontologies to automatically generate training data. Rules or labeling functions (LFs) (defined based on domain knowledge from these sources) are applied on unlabeled data, the output of which is used to train a generative model to generate probabilistic training labels, thus obviating the laborious process of constructing human-annotated labels. Moreover, the LFs can be easily updated when applied to a different institution’s data to incorporate any change in the downstream use case, or to be consistent with the latest domain knowledge with feedback from subject matter experts. This thereby reduces the manual effort of relabeling data based on revised annotation guidelines.

Inspired by this, we use data programming to automatically construct a weakly labeled corpus of radiology reports following our previously proposed Rad-SpatialNet representation schema.^14^ This schema covers spatial information related to both radiological findings and medical devices described in reports. In this, a spatial trigger (eg, spatial preposition, verb) evokes a spatial relation between a finding/device and an anatomical location and forms the lexical unit of a spatial frame. Besides finding, device, and location, the other clinically important contextual information (eg, position status of a device, potential diagnosis) that are associated with a spatial relation acts as participants of a frame and are referred to as spatial frame elements (FEs). A brief description of the spatial trigger and the FEs is presented in Table 1. Available ontologies along with domain language patterns can be easily harnessed through data programming to capture these spatial FEs. Thus, spatial relations in radiology reports are an excellent knowledge-heavy setting to evaluate the data programming paradigm.

**Table 1.** Description of spatial trigger and spatial frame elements defined in the Rad-SpatialNet schema with example

Item	Description	Example
Spatial trigger	The target words (lexical units) of the frames that indicate a spatial relationship exists between a radiological finding and an anatomical location or a medical device and an anatomical location. These are usually prepositions, verbs, and prepositional verbs.	There is hazy opacity **of** the lung consistent with hyaline membrane disease.
Spatial frame elements		
Figure	The object whose location is described through the spatial trigger (usually refers to findings and devices, and sometimes anatomical locations)	There is hazy **opacity** of the lung consistent with hyaline membrane disease.
Ground	The location of the figure described (usually an anatomical structure)	There is hazy opacity of the **lung** consistent with hyaline membrane disease.
Hedge	Uncertainty expressions used by radiologists	There is hazy opacity of the lung **consistent with** hyaline membrane disease.
Diagnosis	Clinical condition or disease associated with a finding suggested as differential diagnoses, usually appears after the hedge-related terms	There is hazy opacity of the lung consistent with **hyaline membrane disease**.
Position status	Any position-related information, usually in context to a medical device	A right PIC catheter **terminates** in the mid SVC.
Relative position	Terms used for describing the orientation of a radiological entity with respect to an anatomical location	The UV line tip is **high** in the right atrium.
Distance	The actual distance of the finding or device from the anatomical location	ETT tube is **1 cm** above the carina.
Reason	Clinical condition or disease that acts as the source of the finding	A subtle area of increased signal adjacent to the left lateral ventricle at the level of corona radiata could be due to a small **lacune**.
Associated process	Any process or activity associated with a spatial relation	During the **movement of the right foot**, there is a small area of cortical BOLD activation adjacent to the area of edema.

*Note*: The **bolded** phrase in the example represents the text that acts as the trigger or the frame element.

**Figure 1.**
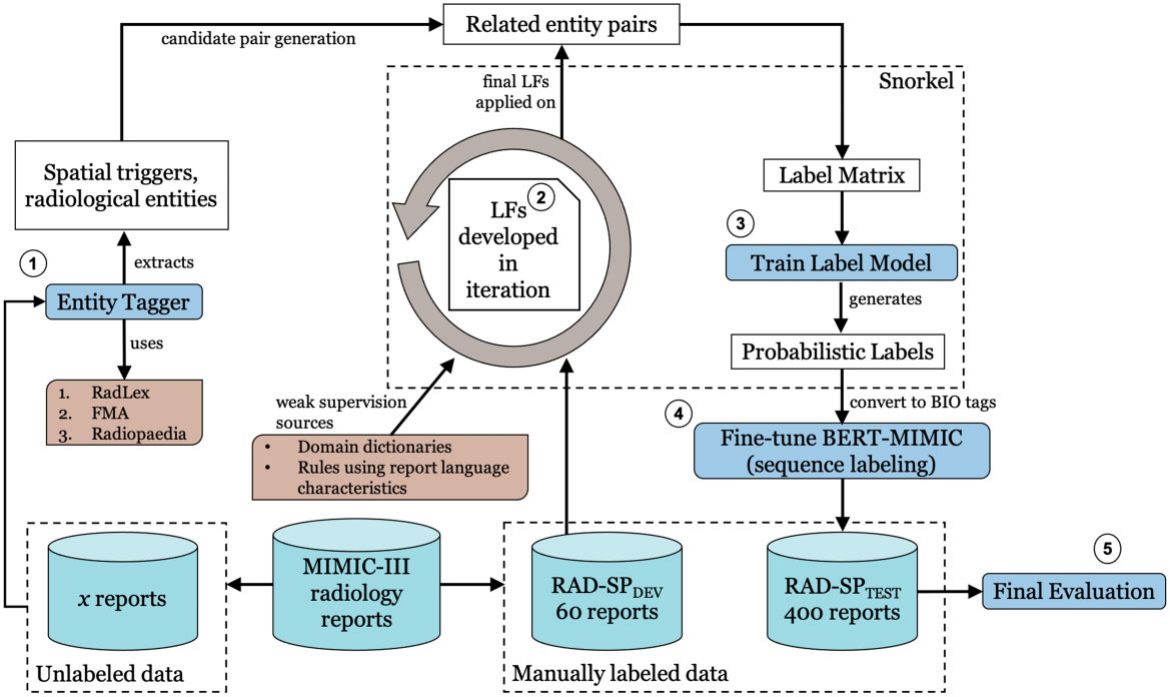
Overview of our weak supervision approach for radiology spatial information extraction. LF: labeling function; BIO: beginning, inside, outside; Rad-SpDev: development set; Rad-Sp_400_: held-out test set. *x* represents the number of unlabeled reports used for training the Label Model (varies from 500 to 50k).

We use the Snorkel framework^5^ to automatically create the weak relation labels. Our LFs are based on the radiology-specific lexicon—RadLex,^15^ regular expressions, language characteristics of report text, and other task-specific heuristics. The generated weak labels are used to train a transformer-based language model, Bidirectional Encoder Representations from Transformers (BERT).^16^ The overall weak supervision pipeline is shown in Figure 1. To assess our approach, we evaluate BERT that is fine-tuned only using weakly labeled reports. We also evaluate sequential fine-tuning performance (fine-tuning on weak labels followed by gold labels) and compare it with a fully supervised variant. The evaluations are performed on 400 radiology reports (comprising of chest X-ray, brain MRI, and babygram reports) annotated in prior work.^14^ We demonstrate that a data programming-based weak supervision method produces promising results in extracting spatial information without using any hand-labeled reports for training apart from relying on a small manually annotated dataset of 60 reports (used for developing LFs and tuning the generative and BERT models). The main implications of this work are:

1. Enabling detailed spatial information extraction from radiology reports without manually labeled training data.
2. Facilitating the generation of more comprehensive weak information labels at large scale to contribute to on-going research in automatically labeling image datasets and other downstream use cases in radiology.
3. Holds potential to extend to different radiology subareas (eg, head CT, knee MRI, etc.) for extracting spatial information

## RELATED WORK

Numerous work has focused on open-domain NLP tasks using weak supervision. Many studies^6–8^^,^^17–20^ have proposed weak supervision methods for named entity recognition, and a few for other tasks such as natural language generation and understanding^21^ and discourse structures.^22^ Recently, there has been increasing work on automatically creating training data and adopting weakly supervised machine learning (ML) methods for NLP tasks in the clinical domain. Wang et al^23^ developed a rule-based NLP method to create labels for training ML models to classify clinical text. Cusick et al^24^ proposed a rule-based approach based on NegEx to generate training labels for identifying current suicidal ideation. Dong et al^25^ adapted a weak supervision approach with rules and contextualized representations to identify rare diseases. Shen et al^26^ adopted a similar weak supervision approach with BERT where they used a rule-based NLP method to automatically generate training labels for classifying lifestyle factors for Alzheimer’s disease. Banerjee et al^13^ proposed a weak supervision method where domain-specific dictionaries are used to heuristically generate training labels to classify evidence of urinary incontinence and bowel dysfunction. Callahan et al^12^ employed data programming and trained LSTM networks for identifying pain-anatomy and implant-complication relations from clinical notes. Peterson et al^11^ trained a BERT model using weakly labeled data generated through data programming to classify relations (eg, severity, stage, etc.) that can be mapped to FHIR representations. Fries et al^9^ utilized data programming with BioBERT to classify medical entities and demonstrated comparable results to fully supervised models on multiple benchmark datasets. Very recently, Humbert-Droz et al^27^ developed a data programming-based weak supervision pipeline using Snorkel to generate weak labels for identifying the presence or absence of symptoms. Moreover, in the biomedical domain, multiple studies have used the Snorkel framework for extracting chemical reaction relationships from biomedical abstracts,^28^ biomedical relation extraction,^29^ and filtering biomedical research articles as relevant or nonrelevant for drug repurposing in cancer.^30^

We see that most studies in the clinical domain use a rule-based approach to create weak labels for binary classification tasks. Differently, our work harnesses the advantages of data programming to generate weak labels for more complex information extraction involving multiple relations. Although Peterson et al^11^ identified different relations associated with a problem description, their approach assumes a single clinical problem in a description whereas our work extracts FEs related to multiple spatial triggers in a report sentence. Moreover, only a few studies^23^^,^^31^^,^^32^ so far have applied weak supervision on radiology report text, 2 of those for binary classification problems (classifying a report as normal vs abnormal^31^ and identifying hip fracture from report^23^) while another^32^ to generate weak anatomical region labels that are subsequently used for training imaging models. Unlike these, this work identifies more detailed information from the reports and thereby generates richer weak labels.

## MATERIALS AND METHODS

### Data

We use 400 (358 containing spatial relations) MIMIC-III^33^ radiology reports (chest X-ray: 136, brain MRI: 127, and babygram: 137) manually labeled as per the Rad-SpatialNet schema^14^ to evaluate our weak supervision pipeline. We refer to this dataset as Rad-Sp_400_. We randomly select a total of 50k unlabeled MIMIC reports (with an almost equal proportion of the 3 report types) to train the generative model and subsequently the weakly supervised BERT_LARGE_-MIMIC model. We manually annotate additional randomly selected 60 MIMIC reports (20 in each of the 3 categories) for building dictionaries, LFs, and hyper-parameter tuning (referred to as Rad-Sp_Dev_). Keeping into account the time and expertise that goes into manual labeling, and that these reports are used for development purposes, these additional annotations are performed by a physician having sound medical knowledge.

### Automatically creating training data

We perform the following sequential steps to programmatically create the weak training labels. For this we employ data programming using the Snorkel framework.^5^

#### Generating candidates for spatially related entities

We identify all the candidate {spatial trigger, radiological entity} pairs where the radiological entity acts as a potential spatial FE with respect to the spatial trigger in a sentence. This involves the following steps:

1. Dictionary construction—We curate 2 dictionaries using preexisting knowledge sources—(1) Rad-Entity_dict_: This contains different types of radiological entities such as radiological findings and anatomical entities using RadLex.^15^ For this, all RadLex terms under the parent RadLex classes *Imaging observation* (RID5), *Clinical finding* (RID34785), *Anatomical entity* (RID3), *Medical device* (RID29033), and *Process* (RID39128) are used for constructing a comprehensive vocabulary representing important radiological entities. Additionally, we also add the terms in Foundational Model of Anatomy (FMA) ontology^34^ to include more anatomical entities and add radiology-specific acronyms and their corresponding expansions from Radiopaedia.^35^ This results in a total of 153 944 terms. (2) Spatial-Trigger_dict_: This contains potential phrases denoting spatial relations between finding/device and location. We develop this by combining the spatial triggers annotated in Rad-Sp_Dev_ to a set of hand-built trigger terms.
2. Expanding Rad-Entity_dict_—We manually add more finding and anatomy-related terms to Rad-Entity_dict_ that are encountered in Rad-Sp_Dev_ but are not present in RadLex or FMA (eg, effacement, volume loss, caudate nucleus head). Based on patterns identified using Rad-Sp_Dev_, we further prepend or append phrases to a set of terms in Rad-Entity_dict_. Specifically, we prepend in 2 ways—(1) prepending phrases such as “area(s) of”, “region(s) of”, and “focus/foci of” to finding-specific terms (eg, hypodensity) and (2) prepending descriptors to certain finding and anatomical entities (eg, prepend “petechial” and “intraparenchymal” to a finding term hemorrhage and prepend combinations of 2 brain lobes such as “frontoparietal” to terms like *lobe* and *cortex*). Finally, we add the plural forms of all terms to the dictionary. For including terms related to “RelativePosition”, “PositionStatus”, and “Hedge” FEs to Rad-Entity_dict_, we construct a list of terms using both Rad-Sp_Dev_ and manually curated terms. Additionally, for “RelativePosition” and “Hedge”, we add all RadLex terms under RadLex class *Location descripto*r (RID5817) and *Certainty descriptor* (RID29), respectively. This increases the total number of terms to 1 492 109.
3. Entity tagging—We apply an entity tagger that extracts all possible text spans in a sentence representing any spatial FE by exactly matching against the terms in Rad-Entity_dict_. For the identified spans having any overlap, the longest span is selected except for a few special cases. Such exceptions include anatomy entities (eg, inferior cerebellar peduncle) that contain location descriptor-related terms (inferior) in which cases we select both inferior and the main anatomical entity cerebellar peduncle as 2 candidate entity spans instead of selecting the longest span. Similarly, candidate spatial triggers are identified using dictionary matching against terms in Spatial-Trigger_dict_. For entities representing “Distance” FE (eg, “*2 mm*”), regular expressions (inspired from Bozkurt et al^36^) are applied for matching. Besides “Distance”, we also develop regular expressions for identifying certain anatomical entities representing segments (eg, “C5-C7”, “T12”).

Finally, all possible {trigger, entity} pairs are generated by combining each identified trigger with all identified radiological entities in a sentence. All these pairs form the candidate spatially related entities.

#### Developing labeling functions

This step involves writing rules or LFs considering both radiology report-specific language characteristics and domain lexicons to vote on a {trigger, entity} pair’s potential FE label (from a set of 9 labels corresponding to 9 spatial FEs). Given a {trigger, entity} pair as input, each LF either assigns an FE label for the pair or abstains (ie, assigns no label). Most LFs include combining dictionary-matching and task-specific heuristics. Dictionaries used in LFs are constructed in a similar manner as for the entity tagging step (described in the above section) for broad categories such as finding, device, and anatomy. Matching against terms in specific dictionaries constrains the semantic type of an entity whereas the task-specific heuristics captures prominent cues to identify the potential spatial role of an entity with respect to a spatial trigger by using linguistic features of a sentence documenting any important clinical information about radiological findings. Examples of heuristics used in LFs to vote a candidate {trigger, entity} pair as “Ground” and “Diagnosis” FE are illustrated in Table 2.

**Table 2.** Heuristics used in 2 sample LFs to label a {trigger (SpTrg), entity (RadEnt)} pair with Ground and Diagnosis frame element relations

FE (features used in LF)	Example sentence	Heuristics
**Ground** (Relative position of RadEnt with respect to SpTrg; semantic type of RadEnt)	The lungs**demonstrate** hazy bilateral opacity of hyaline membrane disease.	SpTrg is any of [*with*|*without*|*show*(*s*)|*demonstrate*(*s*)| *is*|*are*|*reveal*(*s*)] AND RadEnt lies directly adjacent to the left of the SpTrg For other SpTrgs, RadEnt lies to the right of SpTrg AND there is 0–2 words in between SpTrg and RadEnt AND RadEnt is anatomy
**Ground** (Closest SpTrg; semantic type of RadEnt)	There are scattered T2 high signal intensity foci **in** the periventricular white matter and centrum semi-ovale consistent with microvascular angiopathy.	RadEnt lies to the right of SpTrg AND 0 word in between SpTrg and RadEnt AND RadEnt is anatomy For greater than 0 word in between SpTrg and RadEnt, no other trigger in between AND RadEnt is anatomy
**Diagnosis** (Closest SpTrg; semantic type of RadEnt; presence of hedge to the left of RadEnt; RadEnt being the last term)	A patchy area of consolidation is seen **within** the right lower lobe concerning for pneumonia.	RadEnt is finding AND text span to the right of RadEnt is “.” AND IF preposition-containing hedge term between SpTrg and RadEnt ELSE IF a hedge term present to the left of the RadEnt with window length 4 and no additional spatial trigger between SpTrg and RadEnt
**Diagnosis** (Presence of specific hedge-related terms surrounding RadEnt; semantic type of RadEnt)	There is stable opacity **in** the right lower lobe as well as a retrocardiac opacity, these are likely related to atelactases versus pneumonia.	left window = [*represent*, *suggest*, *indicat*, *consistent with*]; right window = [*ruled out*, *excluded*, *vs*, *versus*] any item in left window list present to the left of RadEnt with window length 4 AND RadEnt is finding any item in right window list present to the right of RadEnt with window length 4 AND RadEnt is finding

*Note*: SpTrgs are **bolded** and FEs are underlined.

RadEnt: radiological entity; SpTrg: spatial trigger; FE: frame element; LF: labeling function.

Besides relying on domain ontologies (RadLex, FMA) through dictionary match, the task-specific heuristics are necessary for a complicated task like this where identifying a spatial role (or FE) against a radiological entity is context-dependent (eg, a finding term could be both “Figure” and “Diagnosis” depending on what role it plays in a sentence). This becomes more challenging when there are multiple spatial triggers in a sentence and the same entity is associated with different triggers with different spatial roles (eg, an anatomical entity could be both a “Figure” and “Ground”). Our LFs handle this complexity by considering the position of an entity with respect to a specific spatial trigger in a sentence. We manually examine the sentences in Rad-Sp_Dev_ to build the LFs. The LFs are developed and refined in iteration by evaluating on the annotated Rad-Sp_Dev_ set. We develop 19 LFs in total. The heuristics used in all LFs are included in the Supplementary Table S1.

#### Applying generative model and creating weak labeled data

We use Snorkel’s generative model (known as a Label model) that combines the noisy label outputs from all LFs for a {trigger, entity} pair by estimating the unobserved accuracy of each LF to assign a single probabilistic label for that pair. This generates probablistic training labels (or “weak” labels) for all candidate pairs extracted from the unlabeled report sentences. Since our task is to identify the FEs at the level of each spatial trigger in a sentence, we create separate instance for each trigger by combining all the RadEnts from the {trigger, entity} pairs for which an FE label has been predicted. These modified trigger-level instances are used for further processing.

#### Filtering weak labels

We apply 2 additional constraints to filter the weak labels generated by the Label model to produce a sizable improved weakly labeled training data. First, since “Figure” and “Ground” constitute the 2 fundamental FEs of a spatial frame, we check for the presence of both these FEs among the weak FE label predictions in the trigger-level instances from the above step. Second, we check for the presence of certain frequent phrases surrounding common spatial triggers such as of, with, and in. We ensure that no such phrase is found to the left or right of the trigger. This rule eliminates false positive (FP) triggers (the frequent phrase sets are taken from a previous work^37^). Only if the 2 constraints satisfy, we select that trigger-level instance in our final weak labeled training set.

#### Weakly supervised model—BERT

**Figure 2.**
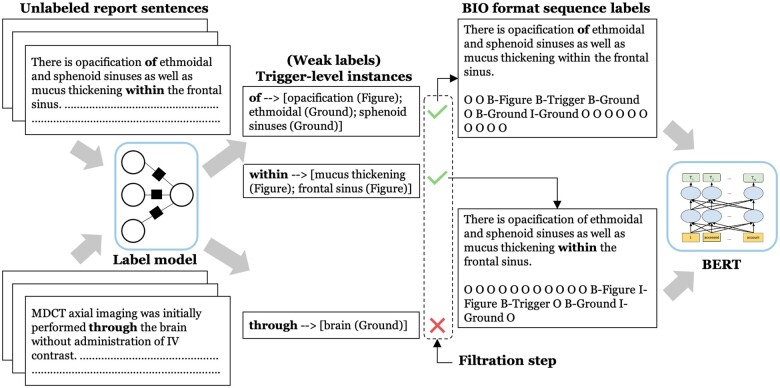
Filtering weak labels and converting the labels to feed into BERT model. All the candidate spatial triggers are shown in **bold**.

We use the final weak labeled training data to fine-tune BERT_LAR__GE_-MIMIC (pretrained on MIMIC notes for 300K steps^38^). We formulate this as a sequence labeling task where we extract spatial FEs provided a spatial trigger in a sentence. For this, we convert each trigger-level instance produced from Label model to a sequence of Beginning-B, Inside-I, and Outside-O (BIO) labels against each word in a sentence. The process of filtering the weak labels and transforming to BIO tag sequence is shown in Figure 2. Each sentence, represented using the standard input sequence format—([CLS] sentence [SEP]), is then fed into BERT. As there can be multiple triggers in a sentence, we mask the words corresponding to a spatial trigger with an identifier $sptrg$to encode the position of a specific trigger. The contextual representations from the BERT encoder output is fed into a linear classification layer to predict label per word.

### Experimental setups

#### Using no gold data

We use varying amounts of unlabeled MIMIC-III radiology reports to generate weak spatial labels and then use these labels to fine-tune the BERT_LARGE_-MIMIC model. Specifically, we use 10%, 25%, 50%, 75%, and 100% of the 50k selected MIMIC reports. We evaluate each variant on the 358 gold annotated test reports (Rad-Sp_400_).

#### Sequential fine-tuning

We perform sequential fine-tuning where we first fine-tune BERT on weakly labeled reports followed by fine-tuning on gold annotated reports (a similar approach that proved to be effective in a recent work by Smit et al^39^ to improve the performance of the automatic rule-based labeler like CheXpert^40^). Specifically, we leverage the best BERT_LARGE_-MIMIC model variant among the 5 variants trained on only weak labels to initialize the model parameters to further fine-tune on gold reports. A total of 358 gold annotated reports are used in this experiment. We report the average F1 measures of a 10-fold cross-validation where 80% of the 358 reports are used for the fine-tuning process, 10% for validation, and 10% for testing. We use the results (average F1 using predicted triggers) of a fully supervised BERT_LARGE_-MIMIC sequence labeling model reported in a previous work^14^ for direct comparison. Note that here we use the same 10-fold split settings as used in the fully supervised experiment in our previous work.

#### Using varying gold data

We also experiment using increasing amounts of gold annotated reports for sequential fine-tuning. Similar to the sequential fine-tuning experiment, the best trained model on weak labels is used to further fine-tune on the gold reports. We use 10% of 358 annotated reports, each for testing and development, and the remaining 80% (ie, 288 reports) for training. Specifically, we use 10%, 25%, 50%, 75%, and 100% of the 288 gold reports for sequential fine-tuning.

### Evaluation

Since our approach involves a pipeline of 2 models (spatial triggers and FEs), to evaluate our weakly supervised BERT model’s end-to-end performance, we consider the spatial triggers that are predicted by the Label model on the test data (Rad-Sp_400_). We apply the 2 constraints (refer section “Filtering weak labels”) to form the model input to BERT. Here, we apply the first constraint with a slight modification (ie, we filter the trigger-level instances for which a Ground FE is predicted by the Label model) in order to increase the recall of spatial triggers as the fine-tuning task uses the trigger positions to predict the associated FEs. We take into account the precision loss related to FEs that are predicted for FP spatial triggers and recall loss related to FEs that are missed for false negative (FN) triggers. These FP and FN triggers are based on the predictions of the Label model on Rad-Sp_400_.

The hyperparameters for both the Label and BERT_LARGE_-MIMIC models are tuned using the 60 annotated reports in Rad-Sp_Dev_ through grid search. For Label model, the number of training epochs, learning rate, L2 regularization, and precision initialization are set at 100, 0.0001, 0.01, and 0.7, respectively. For BERT_LARGE_-MIMIC fine-tuning, the maximum sequence length, learning rate, training epochs are chosen as 128, 2e − 5, and 4, respectively and we use the cased version of the model.

## RESULTS

The coverage of the candidate generation phase is 78.3%, or in other words, our candidate generator identifies 78.3% of the total gold {spatial trigger, radiological entity} pairs from Rad-Sp_400_. The performance measures of the weakly supervised BERT_LARGE_-MIMIC model using increasing amounts of weakly labeled reports are presented in Table 3. We see that the best overall F1 on Rad-Sp_400_ is obtained when 37.5k weakly labeled reports are used. The precision, recall, and F1 measure for identifying the spatial triggers on Rad-Sp_400_ are 70.84%, 75.07%, and 72.89, respectively. We additionally present the results for 500 and 1k weakly labeled reports (in Table 3) to highlight the performance trend even when less than 10% of reports are used in fine-tuning.

**Table 3.** F1 measures of the weakly supervised BERT_LARGE_-MIMIC model on Rad-Sp_400_

Frame elements	# Weak labeled reports for training						
	500	1k	5k	12.5k	25k	37.5k	50k
Ground	54.16	61.90	62.62	63.25	63.53	63.07	63.06
Figure	32.56	41.12	45.47	45.18	44.66	45.93	45.37
Relative position	50.36	50.15	48.34	48.88	49.13	49.22	49.17
Hedge	42.75	41.34	53.65	50.51	51.84	55.00	56.38
Diagnosis	20.77	25.05	29.62	31.23	33.55	33.26	34.12
Position status	43.75	45.08	42.66	38.32	39.88	40.92	41.04
Distance	70.48	64.46	65.49	63.79	66.67	66.67	67.31
Reason	0	0	19.51	27.69	28.12	30.77	31.43
Overall	43.40	49.01	51.64	51.64	51.92	52.47	52.43

*Note*: All the values for the “Associated Process” frame element are zero.

Table 4 shows the sequential fine-tuning results when the model checkpoint corresponding to using 37.5*k* weakly labeled reports are used to further fine-tune on gold reports. We note that the sequential fine-tuning helps to improve the performance for all spatial FEs except for the “Relative Position” FE when compared to the fully-supervised variant. We observe that this improvement is higher for the less frequent FEs (as can be seen from the Count column in Table 4). To demonstrate the effect of increasing size of gold annotated data on the model’s performance, we present the sequential fine-tuning results with varying gold reports in Table 5 on a randomly selected 35 annotated test reports. As expected, the performance of the BERT_LARGE_-MIMIC model improves as the gold data size is increased, however, we observe that the highest or a comparable overall F1 measure is achieved using 75% (ie, 213 reports) of the total annotated reports available for training. Also note that the results for the “Reason” and “Associated Process” FEs are zero in many cases as these are found very infrequently in the dataset.

**Table 4.** Average F1 measures of BERT_LARGE_-MIMIC model over 10-fold CV through sequential fine-tuning (using the model checkpoint obtained after fine-tuning on weak labels of 37.5k reports)

Frame elements	Precision (%)	Recall (%)	F1	FS–F1	Count
Ground	74.64	69.33	71.74	71.51	1537
Figure	71.25	64.90	67.69	65.12	1491
Relative position	65.99	65.18	64.96	66.33	398
Hedge	67.60	61.87	64.04	57.82	249
Diagnosis	58.12	56.14	56.54	50.76	190
Position status	66.18	71.22	68.05	60.37	167
Distance	88.00	90.83	88.36	88.05	45
Reason	53.33	51.29	45.19	0	33
Associated process	60.00	45.00	50.00	0	21
Overall	71.26	66.71	68.76	66.25	3131

FS–F1: fully supervised F1 measures.

**Table 5.** Sequential fine-tuning results (F1 measures) of BERT_LARGE_-MIMIC (using the model checkpoint obtained after fine-tuning on weak labels of 37.5k reports) on randomly selected 35 test reports with increasing amount of gold reports used in the fine-tuning process

Frame elements	% of gold reports used				
	10	25	50	75	100
Ground	64.52	68.09	66.32	66.31	67.38
Figure	51.72	54.75	56.82	59.55	57.78
Relative position	53.66	60.47	58.54	55.00	55.81
Hedge	55.00	68.18	76.92	76.92	73.17
Diagnosis	48.48	54.55	53.33	70.97	62.50
Position status	54.55	61.54	61.54	57.14	66.67
Distance	40.00	33.33	66.67	57.14	75.00
Associated process	0	0	100	100	100
Overall	56.05	60.43	62.03	63.62	63.01

*Note*: All the values for the “Reason” frame element are zero. One hundred percent corresponds to 288 gold reports.

## DISCUSSION

We develop a weakly supervised pipeline based on data programming technique to extract spatial relations from radiology text. This is an early attempt to automatically create weak labels in the radiology domain covering detailed and important spatial information of clinical importance that could be used for various clinical informatics applications, unlike the 3 previous studies that employed weak supervision for simpler binary classification and anatomy tagging.^23^^,^^31^^,^^32^ The results in Table 3 demonstrate that our proposed pipeline performs decently given the complexity of information extracted and without any reliance on the time-consuming and expensive manual labeling process. Although they do not surpass the fully supervised model’s performance, they hold the potential to identify a variety of important clinical information without using any hand-labeled training data. Further, our findings on sequential fine-tuning (Table 4) also reflect the advantages of leveraging a MIMIC pretrained model first fine-tuned on domain and task specific data (weakly labeled radiology reports) and then on gold annotated data instead of just fine-tuning on the gold data. This is in line with the findings demonstrated for the CheXbert model where combining the annotations of a rule-based labeler and expert annotations resulted in better performance.^39^ Additionally, Table 4 indicates that leveraging weak supervision boosts the performance in identifying rare FEs.

In general, there is a major trend in NLP (both open-domain and clinical) at the moment focusing on models that work on small amounts of data. Few-shot learning (eg, with prompts) is one option, weak supervision is another, each with pros/cons (weak supervision allows for more direct injection of knowledge). It is scientifically critical that these options get fleshed out since different methods may be more appropriate under different conditions.

Our weak supervision approach provides sufficient flexibility as the LFs can be fairly easily modified to incorporate any change in reporting style and other institutional reports.

For example, let us consider the following 2 reporting styles:

1. There are calcified atherosclerotic changes in the brain parenchyma.
2. Brain parenchyma: There are calcified atherosclerotic changes.

While the first style is more commonly encountered in radiology reports to describe findings (eg, atherosclerotic changes) and their locations (eg, brain parenchyma), some institutions or radiologists may prefer the second format (ie, location: findings). Such changes in reporting format may necessitate some updates in the LFs which could be easily incorporated as and when needed. Moreover, as new FEs are added to the representation schema for different downstream use cases, we can add additional LFs to cover those. Additionally, as the LFs are developed using the more general language characteristics of radiology text and the finding/anatomy dictionaries are primarily based on standard medical ontologies (eg, FMA), they are mostly generalizable, that is, they hold potential in identifying the spatial FEs belonging to different imaging modalities and human anatomies (beyond the 3 report types used in this work). For instance, our LFs will be able to identify spatial information from a pelvic ultrasound report sentence as well (eg, identifying the finding leiomyomas and its location *uterus* from the sentence—“Multiple leiomyomas in the uterus.”). This is not explored in this work, however, we plan to do a thorough analysis to examine the performance of our weak supervision pipeline when applied to reports of multiple institutions, modalities, and anatomies in the future.

Although the dictionaries we developed in this work are comprehensive enough (at least for the 3 radiology subareas considered in this article), we intend to further improve the coverage of the candidate generation step that generates the candidate {spatial trigger, radiological entity} pairs by further expanding the terms in the dictionaries that can detect more variation of radiological entities. The coverage is mainly impacted because of misspellings and the dictionaries lacking less common phrase variations representing findings and anatomies. Some of such phrases include findings such as gastric distention, gross formational abnormalities, low attenuation structure, signal gap, and mesenteric stranding and anatomical locations such as portal vein, deep venous system, mediastinal margin, cavernous carotid, and antecubital fossa. This also reflects the challenges involved in creating more robust dictionaries as there are different and many possible ways of expressing radiological entities and we leave this to future work.

## CONCLUSION

We propose a data programming-based weak supervision method to identify spatial triggers and spatial FEs from radiology reports. The performance in extracting the triggers and elements are satisfactory, with F1 measures of 72.89 and 52.47, respectively. This is achieved without using any manually labeled reports for training. Our results also indicate that sequential fine-tuning using MIMIC pretrained BERT model, first on weakly labeled reports and then on gold reports results in better performance compared to a fully supervised MIMIC BERT model.

## Supplementary Material

ooad027_Supplementary_Data

## Data Availability

The instructions to access the weakly labeled dataset can be found at: https://github.com/krobertslab/datasets/tree/master/rad-weak-supervision.
