# Supplementary material for: Weakly supervised spatial relation extraction from radiology reports
Source: JAMIA Open. 2023 Apr 22;6(2):ooad027. doi: 10.1093/jamiaopen/ooad027 (PMC10122604; doi:10.1093/jamiaopen/ooad027)
Supplement: ooad027_Supplementary_Data [file ooad027_supplementary_data.docx]

**SUPPLEMENTARY MATERIAL**

**Table S1:** Heuristics used in the labeling functions to identify the spatial frame elements. LF - Labeling Function. RadEnt - Radiological Entity. SpTrg - Spatial Trigger.

| **Spatial Frame Element** | **Heuristics** |
| --- | --- |
| **Figure (LF 1)** | anatomies to ignore = [*side, region, portion, part, territory, fragment, margin, site, aspect, division, area, branch*]  anatomy related terms = all anatomies - anatomies to ignore  RadEnt is neither relative position nor position status nor hedge AND   - IF SpTrg is any of [*with*\|*without*\|*show*(*s*)\|*demonstrate*(*s*)\|*is*\|*are*\|*reveal*(*s*)] AND RadEnt lies to the right of SpTrg AND RadEnt is finding - ELSE IF RadEnt lies to the left of SpTrg AND any of [*with tip*\|*with its tip*\|*with the tip*] does not follow RadEnt AND any of [*tip of*\|*tip of the*] does not precede RadEnt AND - IF no preposition-containing hedge term between SpTrg and RadEnt AND the other trigger term between SpTrg and RadEnt is ‘*of*’ AND RadEnt is ‘*tip*’ - ELSE IF no preposition-containing hedge term between SpTrg and RadEnt AND no additional spatial trigger between SpTrg and RadEnt AND RadEnt is not an anatomy-related term - ELSE IF 0 word in between SpTrg and RadEnt AND RadEnt is not an anatomy-related term |
| **Figure (LF 2)** | - RadEnt lies to the left of SpTrg AND RadEnt belongs to acronyms dictionary |
| **Figure (LF 3)** | specific terms = [*collapsed*, *engorged*, *widened*, *calcified*, *unfolded*, *occluded*, *prominent*, *inflated*, *hypoinflated*, *hyperinflated*, *aerated*, *hyperaerated*, *hypoaerated*, *narrowed*]   - RadEnt lies to the right of SpTrg AND SpTrg is any of [*is*\|*are*] AND RadEnt belongs to specific terms list |
| **Ground (LF 1)** | - SpTrg is any of [*with*\|*without*\|*show*(*s*)\|*demonstrate*(*s*)\|*is*\|*are*\|*reveal*(*s*)] AND RadEnt lies directly adjacent to the left of the SpTrg - For other SpTrgs, RadEnt lies to the right of SpTrg   AND there is 0-2 words in between SpTrg and RadEnt AND RadEnt is anatomy |
| **Ground (LF 2)** | RadEnt lies to the right of SpTrg AND   - 0 word in between SpTrg and RadEnt AND RadEnt   is anatomy   - For greater than 0 word in between SpTrg and RadEnt,   no other trigger in between AND RadEnt is anatomy |
| **Ground (LF 3)** | *Regular expressions used for matching specific anatomy patterns*:  anatomy with segment = "[A-Z][0-9]{1,2}[\-\|\/][A-Z]?[0-9]{1,2}"  anatomy with segment without hyphen = "[A-Z][0-9]{1,2}"  anatomy segment body = "[A-Z][0-9]{1}\s{1,}body"  RadEnt lies to the right of SpTrg AND   - 0 word in between SpTrg and RadEnt AND RadEnt   matches any of the three anatomy patterns   - For greater than 0 word in between SpTrg and RadEnt,   no other trigger in between AND RadEnt matches any of the three anatomy patterns |
| **Ground (LF 4)** | specific diseases = [*hmd, hyaline membrane disease, ards, acute respiratory distress syndrome, rds, respiratory distress syndrome*]   - RadEnt lies to the right of SpTrg AND RadEnt belongs to acronyms dictionary except for the terms in specific diseases list |
| **Diagnosis (LF 1)** | RadEnt is finding AND text span to the right of RadEnt  is ‘.’ AND   - IF preposition-containing hedge term between SpTrg and RadEnt - ELSE IF a hedge term present to the left of the RadEnt with window length 4 and no additional spatial trigger between SpTrg and RadEnt |
| **Diagnosis (LF 2)** | left window = [*represent*, *suggest*, *indicat*, *consistent* *with*];  right window = [*ruled* *out*, *excluded*, *vs*, *versus*]   - any item in left window list present to the left of RadEnt with window length 4 AND RadEnt is finding - any item in right window list present to the right of   RadEnt with window length 4 AND RadEnt is finding |
| **Diagnosis (LF 3)** | specific diseases = [*hmd, hyaline membrane disease, ards, acute respiratory distress syndrome, rds, respiratory distress syndrome*]   - RadEnt lies to the right of SpTrg AND RadEnt belongs to specific diseases list AND text span to the right of RadEnt is ‘.’ |
| **Diagnosis (LF 4)** | - RadEnt lies to the right of SpTrg AND no additional spatial trigger or hedge term to the right of RadEnt AND RadEnt is finding |
| **Diagnosis (LF 5)** | RadEnt lies to the right of SpTrg AND RadEnt is finding   - IF preposition-containing hedge term between SpTrg and RadEnt - ELSE IF a hedge term present between SpTrg and RadEnt and no additional spatial trigger between SpTrg and RadEnt |
| **Hedge (LF 1)** | - RadEnt lies to the right of SpTrg AND RadEnt is a hedging-related term AND a finding term is present to the right of the RadEnt |
| **Hedge (LF 2)** | RadEnt lies to the left of SpTrg AND RadEnt is a hedging-related term AND   - 0 word in between SpTrg and RadEnt OR a finding term is present between SpTrg and RadEnt |
| **Distance (LF 1)** | device related = [*tube, catheter, ett, tip, port, lead, device, drain, screw*]  *Regular expressions used for matching distance-related entities*:  distance first pattern = (\d+\.( )?\d+\|\d+( )?\.\d+\|\.\d+\|\d+) *([\- ](mm\|cm\|millimeter(s)?\|centimeter(s)?)(?![a-z/]))  distance second pattern = (\d+\.( )?\d+\|\d+( )?\.\d+\|\.\d+\|\d+) *(\-? *(mm\|cm\|millimeter(s)?\|centimeter(s)?)(?![a-z/]))  distance third pattern = \b(few\|some)\b\s{1,}\b(mm\|mms\|millimeter\|millimeters\|cm\|cms\|centimeter\|centimeters)\b   - any of the terms in device related list is present in the sentence containing RadEnt AND RadEnt matches any of the three distance patterns |
| **Position Status (LF 1)** | device related = [*tube, catheter, ett, tip, port, lead, device, drain, screw*]   - RadEnt is a position status-related term AND any of the terms in device related list is present in the sentence containing RadEnt |
| **Relative Position (LF 1)** | - RadEnt is a relative position-related term AND the next or preceding word of RadEnt is contained in any of the terms in anatomies dictionary |
| **Reason (LF 1)** | - RadEnt is finding AND any reason associated hedge term is present to the left of RadEnt with window length 4 |
| **Associated Process (LF 1)** | - 0 word in between SpTrg and RadEnt AND RadEnt is an associated process-related term - For greater than 0 word in between SpTrg and RadEnt, no other trigger in between AND RadEnt is an associated process-related term |
